# 

**DOI:** 10.1192/bjb.2018.104

**Published:** 2019-08

**Authors:** Rebecca Smith

**Affiliations:** Pallant Medical Chambers West Kent, Forum House, Stirling Road, Chichester, West Sussex, PO19 7DN, UK. Email: rebecca.smith103@nhs.net

This is an insightful and extremely valuable book depicting a man's journey through depression and anxiety. It is based on Brent Williams’ experiences and is a deeply personal account, but is also based on sound medical science. Williams' personal experiences and the unusual graphic novel format make the book very accessible, allowing the reader to understand the experience of depression and anxiety. It is beautifully illustrated by Korkut Öztekin, with pictures which often ‘say’ so much more than words in terms of evoking the atmosphere and man's mood.

We journey with the man from a period of deep depression to recovery, passing through multiple steps on the way– including trying to recover alone, resisting help and later becoming open to help. The book offers an explanation as to how and why one might be feeling depressed and anxious, as well as enabling the reader to learn about the symptoms of depression and anxiety.

Importantly, the story shows the reader how to take the steps towards recovery. The man is told, ‘[y]ou need to break the downward spiral … by doing lots of small and manageable things’. The reader is exposed to breathing techniques, mindfulness and the importance of nature, learning, human contact and exercise amongst other practical steps which help promote recovery.

The book also demonstrates a realistic recovery path with the ups and downs which are so typical. Most significantly, by charting the man's recovery it offers hope and demonstrates a way forward which will be helpful and comforting to those with similar problems. There are elements of the narrative which are perhaps a little alien to the National Health Service culture. Nevertheless, this is a much-needed book which will be very useful to patients and their families/friends as well as a great help to general practitioners by supporting the messages we give.

